# Psychological impact of Covid-19 pandemic on oncological patients: A survey in Northern Italy

**DOI:** 10.1371/journal.pone.0248714

**Published:** 2021-03-16

**Authors:** Eva Pigozzi, Daniela Tregnago, Lucia Costa, Jessica Insolda, Enrico Turati, Michela Rimondini, Valeria Donisi, Pietro Madera, Francesco Fiorica, Jacopo Giuliani, Filippo Greco, Anna Mercanti, Annarita Trolese, Lara Furlani, Paolo Piacentini, Emilia Durante, Marta Mandarà, Sara Pilotto, Alice Avancini, Ilaria Trestini, Marta Zaninelli, Francesca Moretti, Michele Milella, Andrea Bonetti

**Affiliations:** 1 Department of Medical Oncology, AULSS 9 Scaligera, Verona, Italy; 2 Section of Oncology, University of Verona School of Medicine, Verona University Hospital Trust, Verona, Italy; 3 Clinical Psicology Unit, AULSS9 Scaligera, Verona, Italy; 4 Department of Lifesciences and Biotechnology, University of Ferrara, Ferrara, Italy; 5 Clinical Psicology Unit, Verona University Hospital Trust, Verona, Italy; 6 Department of Radiation Oncology and Nuclear Medicine, AULSS 9 Scaligera, Legnago, VR, Italy; 7 Biomedical, clinical and experimental sciences, Department of Medicine, University of Verona, Verona, Italy; 8 Medical Direction, AULSS 9 Scaligera, Legnago, VR, Italy; National Institute for Infectious Diseases Lazzaro Spallanzani-IRCCS, ITALY

## Abstract

The psychological impact of the Covid 19 pandemic on cancer patients, a population at higher risk of fatal consequences if infected, has been only rarely evaluated. This study was conducted at the Departments of Oncology of four hospitals located in the Verona area in Italy to investigate the psychological consequences of the pandemic on cancer patients under active anticancer treatments. A 13-item *ad hoc* questionnaire to evaluate the psychological status of patients before and during the pandemic was administered to 474 consecutive subjects in the time frame between April 27^th^ and June 7^th^ 2020. Among the 13 questions, 7 were considered appropriate to elaborate an *Emotional Vulnerability Index* (*EVI*) that allows to separate the population in two groups (low versus high emotional vulnerability) according to observed median values. During the emergency period, the feeling of high vulnerability was found in 246 patients (53%) and was significantly associated with the following clinical variables: female gender, being under chemotherapy treatment, age ≤ 65 years. Compared to the pre-pandemic phase, the feeling of vulnerability was increased in 41 patients (9%), remained stably high in 196 (42%) and, surprisingly, was reduced in 10 patients (2%). Overall, in a population characterized by an high level of emotional vulnerability the pandemic had a marginal impact and only a small proportion of patients reported an increase of their emotional vulnerability.

## Introduction

On December 2019, an outbreak of novel coronavirus disease (COVID-19) occurred in Wuhan, linked to the severe adult respiratory syndrome coronavirus 2 (SARS-CoV-2). It is characterized by rapid human to human transmission from droplet contamination **[1, 2]**. The infection spread from China to Europe and to the rest of the world, becoming rapidly pandemic; as of June 21^st^ 2020, the World Health Organization reported 8,708,008 confirmed cases, with 461,715 deaths, world-wide.

In Europe, Italy was severely hit, especially the Lombardia and bordering regions such Piemonte, Emilia Romagna and Veneto, with 238,275 cases and 34,610 deaths reported, representing a global share of 2.7% and 7.5% for incidence and mortality, respectively **[3]**.

To contrast the infection the Italian Government issued a series of ordinances gauged on the risk of infection. In phase 1 (from March 9^th^ to May 3^rd^) **[4]** a general lockdown was enforced, during which most industrial and commercial activities were suspended and people were asked to stay at home and to leave home only to satisfy primary needs (to buy food, personal hygiene items, house cleaning supplies, etc); the majority of hospitals, especially in Northern Italy, became hostages of the pandemia and many patients died leaving a strong impact not only to sick people but to the entire population. In this catastrophic emergency the health system had to adapt to meet the needs of patients infected, quite often very sick, while maintaining essential healthcare for all. Furthermore, emphasis was put on the need to ensure essential care for patients with cancer, a potentially frailer fringe of the population, exposed to both a higher risk of COVID-19 and fatal consequences **[5–7]**. During the following phases (phase 2 from May 04^th^ to June 14^th^ and phase 3 which started on June 15^th^) industrial and commercial activities were gradually reopened and people regained their right to move, but still respecting some rules such as physical distancing and wearing a facial mask.

Given the high infection rate of SARS-Cov-2, activities in favor of cancer patients were remodulated to ensure that patients were not exposed to COVID-19. Face-to-face consultations were, whenever possible, taking place via web consulting or by telephone calls. Patients with non-urgent appointments that would require them to be physically present in the hospital for routine and follow-up visits or surgeries would be postponed as often as possible. In addition, the centers did not allow visitors or caregivers to accompany their loved ones when admitted to the hospital for infusions or radiation treatment, as visitors could potentially be (unknowingly) COVID-19 positive. Patients with mild symptoms consistent with COVID-19 were told not to come for their appointments and to follow national guidance on isolation and/or quarantine **[8, 9]**.

These directives were taken very seriously by patients and by mid-April 2020 the decision to keep away from the hospital all the cancer patients not in need of active therapy resulted in a reduction not only of visits (estimated around 15–20%) but also in a drop in the number of new diagnosis of solid tumors in the same range **[10–12]**.

Research on the psychological impact of COVID-19 on cancer patients is still sparse, but it is conceivable that this pandemic should have a negative impact on the feeling of vulnerability in this population, although a recent study showed that when cancer patients are supported by a health care team which includes psychologists experience a better quality of life **[13]**.

This study was planned to investigate the psychological consequences of the current pandemic on patients dealing with a serious oncological disease and under active anticancer treatments at the Departments of Oncology of the University Hospital of Verona and of the Health Maintenance Organization “Scaligera” of the Veneto Region, in Northern Italy.

## Methods

### Study design and participants

This prospective study was conducted at the Departments of Oncology of the University Hospital in Verona and at the Department of Oncology of the Health Maintenance Organization “Scaligera” of the Veneto Region (which includes the Cancer Centers of the Hospitals in Legnago, San Bonifacio and Villafranca). Clinical data were retrospectively retrieved from the medical records, including demographic and clinical characteristics. This study was approved by the Ethics Committee of Verona and Rovigo Provinces on April 16^th^ 2020 and all the enrolled patients signed a written inform consent, no minors have been included in the study.

Overall 474 questionnaires were administered. For each patient, sociodemographic characteristics (age, gender, marital, education and occupancy status) and cancer history (primary tumor diagnosis, stage, line, setting, type of therapy and performance status) were collected.

### Scoring of COVID-19 psychological impact

The psychosocial impact of Covid-19 on patients affected by solid and hematologic malignancies under active treatments was evaluated through a 13-item *ad hoc* questionnaire (**Table 1**) prepared by the clinical Psychologists of our group and submitted to patients by staff members in the time frame between April 27^th^ and June 7^th^.

**Table 1:** Questionnaire *for Emotional Vulnerability Index* (*EVI*) evaluation.

Question n.	Description
**1**	Did you result positive to the Covid 19 test?
**2**	Did a member of your family or a friend result positive to the Covid 19 test?
**3**	How much do you feel anxious/worried for your cancer?(before and during the pandemia)
**4**	How much do you feel sad/discouraged for your cancer? (before and during the pandemia)
**5**	How much do you feel fragile/vulnerable for your cancer?(before and during the pandemia)
**6**	Are you pessimistic about the cure of your cancer? (before and during the pandemia)
**7**	How much do you feel disoriented/confused about the management of your cancer? (before and during the pandemia)
**8**	How much the concerns about your cancer influence the quality of your sleep?
**9**	Do you feel pleasure for actions you have always enjoyed?
**10**	How much do you feel supported/helped in dealing with your cancer by your family members?
**11**	How much do you feel supported/helped in dealing with your cancer by the staff of the oncology center?
**12**	Overall, in this situation of generalized hardship due to Covid 19 emergency do you feel your discomfort increase?
**13**	How much the Covid 19 emergency influences the management of your cancer

For questions 1 and 2 the answers could be Yes/No; for questions 3–13 the answers could be graded according to a numeric scale 1–2 (not at all, a little); 3–4 (quite, a lot) and patients were asked to score their feelings before and during the pandemic.

**Fig 1:**
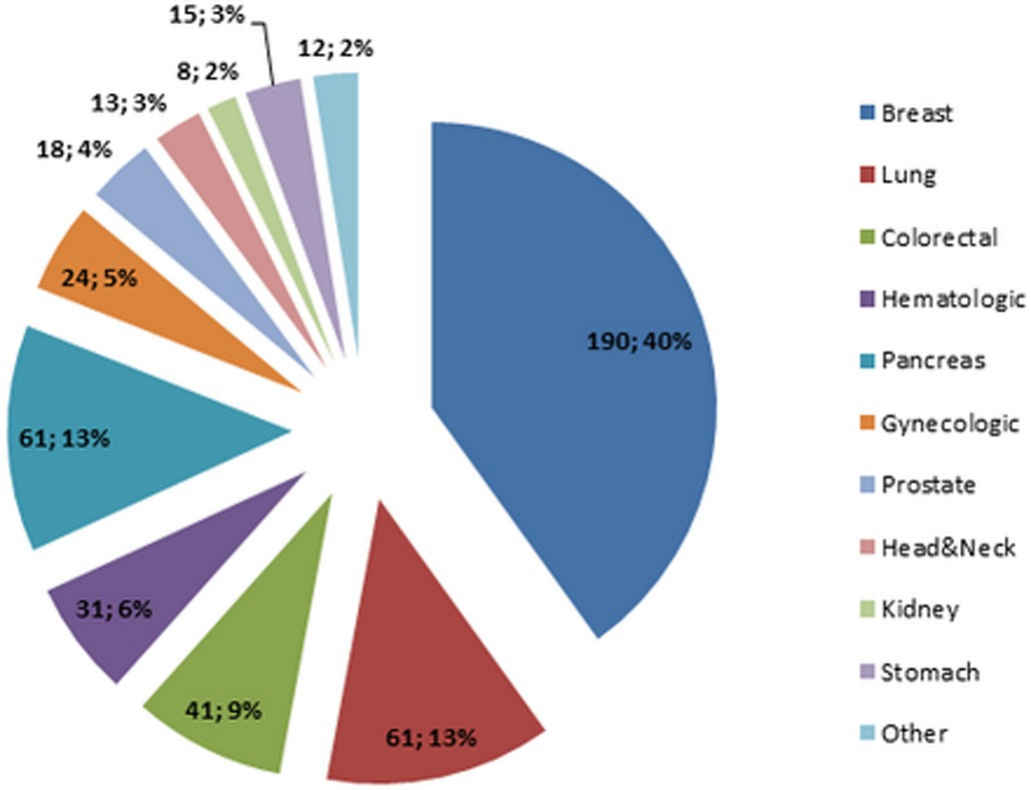
Types of cancers among the included patients.

For questions 1 and 2 the answers could be Yes/No while for questions 3–13 the answers could fall in a 4-point Likert scale in which 1 and 2 means “not at all, a little” while 3–4 means “quite, a lot”; for questions 3 to 13 patients were asked to give a score to their feeling before and during the pandemic. Furthermore, among the 13 questions, psychologists identified the seven questions included from n. 3 to n. 9 as crucial for the classification of emotional vulnerability (i.e. main anxiety /depression symptoms). By summing the scores given by the patient to each of these questions a *Emotional Vulnerability Index* (*EVI*) was obtained (score range 7–28). Scores were classified on the basis of the median grade: low *EVI* < median; moderate/high ≥ the median. To investigate the impact of the Covid 19 pandemic on the psychological status of patients the respondents were classifieds in 4 categories according to the observed median value: low/low (score < the median value pre and during Covid19 outbreak), low/high (score < the median value pre, ≥ the median value during Covid19 outbreak), high/low (score ≥ the median value pre, < the median value during Covid19 outbreak), high/high (score ≥ the median value pre and during). The quartile distribution of the scores pre and during the pandemic is shown in Fig 1.

### Statistical analysis

For descriptive analysis, variables are presented as number and percentage (%). We used the Chi-square test (significance α = 0,05) to evaluate whether there was any correlation between clinic-demographic factors and the *EVI* among oncological patients. Clinical and demographic factors included were age (18–65 and over 65), gender, Performance status (ECOG 0–1 versus 2 or higher), stage (I-III versus IV), therapy setting (neo-adjuvant/adjuvant versus metastatic), therapy line (front line versus subsequent), type of therapy (chemotherapy versus biologicals) and the pandemic organizational phase (phase 1 versus phase 2).

## Results

### Demographic and clinical characteristics

Overall, 474 patients were enrolled and completed individual questionnaires. Twelve questionnaires were incompletely filled. **Table 2** lists the main clinical and demographic characteristics of patients.

**Table 2:** Clinical and demographic characteristics of patients.

Characteristics	Total		Breast		Lung		Colorectal		Gynecologic		Hematologic		Stomach		Head & Neck		Prostate		Pancreas		Kidney		Other	
	N	(%)	N	(%)	N	(%)	N	(%)	N	(%)	N	(%)	N	(%)	N	(%)	N	(%)	N	(%)	N	(%)	N	(%)
**Age at assessment**																								
18–40	30	6	16	8	3	5	0	0	1	4	4	13	0	0	1	8	0	0	3	5	0	0	2	17
41–65	260	55	115	60	26	43	26	63	14	58	12	39	8	53	7	54	7	39	35	57	3	38	7	58
>65	184	39	59	31	32	52	15	37	9	38	15	48	7	47	5	38	11	61	23	38	5	63	3	25
**Gender**																								
Female	309	65	188	98	22	36	22	54	24	100	10	32	6	40	2	15	0	0	27	44	2	25	8	67
Male	165	35	2	1	39	64	19	46	0	0	21	68	9	60	11	85	18	100	34	56	6	75	4	33
**Marital status**																								
Single	43	9	16	8	3	5	4	10	2	8	3	10	1	7	1	8	2	11	8	13	1	13	2	17
Married or domestic partnership	378	80	153	80	48	79	36	88	17	71	24	77	13	87	12	92	15	83	45	74	7	88	8	67
Widowed	40	8	17	9	9	15	1	2	2	8	3	10	1	7	0	0	0	0	6	10	0	0	1	8
Divorced/Separated	13	3	4	2	1	2	0	0	3	13	1	3		0	0	0	1	6	2	3	0	0	1	8
**Education status**																								
Elementary school	57	12	14	7	14	23	5	12	3	13	8	26	0	0	1	8	0	0	9	15	2	25	1	8
Secondary school graduate	138	29	56	29	20	33	12	29	9	38	8	26	7	47	7	54	5	28	10	16	2	25	2	17
High school graduate	174	37	67	35	16	26	14	34	11	46	12	39	6	40	4	31	9	50	24	39	4	50	7	58
Graduated	105	22	53	28	11	18	10	24	1	4	3	10	2	13	1	8	4	22	18	30	0	0	2	17
**Employment status**																								
Employed	159	34	74	39	18	30	15	37	5	21	10	32	2	13	4	31	6	33	16	26	2	25	7	58
Unemployed	49	10	21	11	4	7	4	10	8	33	1	3	2	13	1	8	1	6	6	10	1	13	0	0
Retired	230	49	81	42	36	59	19	46	11	46	18	58	5	33	7	54	11	61	29	48	5	63	3	25
Other (student, job-seeker.)	36	8	14	7	3	5	3	7	0	0	2	6	6	40	1	8	0	0	10	16	0	0	2	17
**Diagnosis**																								
2019–2020	296	62	106	55	45	75	29	71	15	62	17	55	12	80	9	69	11	61	47	77	3	38	7	58
2017–2018	73	15	26	14	9	15	7	17	4	17	6	19	1	7	0	0	4	22	10	16	3	38	0	0
2016 or before	105	22	58	30	7	11	5	12	5	21	8	26	2	13	4	31	3	17	4	7	2	25	5	42
**ECOG PS**																								
0–1	444	94	184	96	56	92	39	95	22	92	30	97	14	93	11	85	16	89	54	89	8	100	11	92
≥2	30	6	6	3	5	8	2	5	2	8	1	3	1	7	2	15	2	11	7	11	0	0	1	8
**Stage**																								
I	81	17	43	23	4	7	2	5	2	8	15	48	2	13	2	15	2	11	8	13	0	0	1	8
II	87	18	37	19	12	20	13	32	3	13	2	6	2	13	2	15	6	33	8	13	0	0	2	17
III	77	16	27	14	9	15	2	5	10	42	11	35	1	7	4	31	2	11	10	16	0	0	1	8
IV	229	48	83	43	36	59	24	59	9	38	3	10	10	67	5	38	8	44	35	57	8	100	8	67
**Anticancer therapy**																								
Chemotherapy	291	61	108	57	38	62	35	85	16	67	15	48	12	80	9	69	8	44	40	66	1	13	9	75
Target therapy	109	23	56	29	9	15	4	10	8	33	4	13	2	13	2	15	4	22	14	23	6	75	0	0
Immunotherapy	47	10	7	4	14	23	2	5	0	0	12	39	1	7	2	15	0	0	7	11	1	13	1	8
Radiotherapy only	14	3	11	6	0	0	0	0	0	0	0	0	0	0	0	0	3	17	0	0	0	0	0	0
Endocrine therapy	13	3	8	4	0	0	0	0	0	0	0	0	0	0	0	0	3	17	0	0	0	0	2	17
**Setting of therapy**																								
Neoadjuvant/Adjuvant/Early or Locally Advanced	245	52	109	57	25	41	17	41	15	63	0	0	0	0	8	61	10	56	26	42	0	0	4	33
Metastatic 1L	124	26	48	25	19	31	13	32	3	13	23	74	10	67	3	23	3	17	17	28	4	50	5	42
Metastatic 2L	60	13	17	9	10	16	7	17	5	21	6	19	4	27	1	8	3	17	9	15	2	25	3	25
Metastatic 3L	19	4	6	3	5	8	1	2	1	4	1	3	0	0	0	0	1	6	3	5	1	13	0	0
Metastatic subsequent Lines	26	5	11	6	2	3	3	7	0	0	1	3	1	7	1	8	1	6	6	10	1	13	0	0
**Hospital**																								
AULSS9 Scaligera- Mater Salutis	177	37	66	35	24	39	11	27	14	58	22	71	6	40	9	69	10	56	1	2	8	100	6	50
AULSS9 Scaligera- Fracastoro	50	11	24	13	1	2	9	22	2	8	9	29	1	7	0	0	1	6	1	2	0	0	2	17
AULSS9 Scaligera- Magalini	7	1	3	2	1	2	1	2	0	0	0	0	0	0	0	0	1	6	0	0	0	0	1	8
AOUI Ospedale Civile Maggiore	133	28	58	30	22	36	14	34	1	4	0	0	5	33	2	15	2	11	26	43	0	0	3	25
AOUI Policlinico G.B.Rossi	107	23	39	20	13	21	6	15	7	29	0	0	3	20	2	15	4	22	33	54	0	0	0	0

The majority of patients were female (309, 65%) and median age was 62 (20–97). Three hundred and seventy eight patients (80%) were married or in a domestic partnership, forty three (9%) were single, forty (8%) were widowed and thirteen (3%) were divorced/separated. The majority of enrolled patient (174, 37%) held a university degree, 138 (29%) have a high school diploma, one hundred and five (22%) have a primary school diploma while the remaining fifty seven (12%) did not go beyond the elementary school.

Among respondents, the most common oncological diagnoses were breast cancer (190 patients, 40%), lung cancer (61 patients, 13%), pancreatic cancer (61 patients, 13%), colorectal cancer (41 patients, 8%), hematologic malignancies (31 patients, 6%), gynecologic cancer (24 patients, 5%), prostate cancer (18 patients, 4%), stomach cancer (15 patients, 3%), head and neck cancer (13 patients,3%), kidney cancer (8 patients, 2%) and other such melanoma, sarcoma and testicular cancer (11 patients, 3%) (**Fig 1)**.

Metastatic disease (stage IV) was the most prevalent condition (229 patients, 48%); chemotherapy was the prevalent type of treatment for the majority of patients(291, 61%); targeted therapy was given to 109 patients (23%), while 47 patients (10%), 14 patients (3%) and 13 patients (3%) were receiving immunotherapy, radiotherapy or endocrine therapy, respectively.

### Questionnaire results

No patient reported Covid 19 positivity, while 11 of their relatives had had a positive test. The vast majority of patients reported a strong family support (question n. 10), with 429 subjects (90%) grading such support as “quite/a lot”. Similar results were obtained regarding support received by cancer center healthcare providers, graded as 3–4 by 90% of patients. However, the situation of generalized hardship due to the Covid 19 emergency increased the patient’s discomfort “quite/a lot” (question n. 12) in a sizable number of patient (165, 35%). Regarding the *Emotional Vulnerability Index*, the results for the pre-emergency period show low level of emotional distress. In fact only a minority of patients (187, 39%) gave on average to these questions a 3–4 score meaning that they were not able to cope with their cancers (**Table 3**).

**Table 3:** Variables for *EVI* evaluation pre and during the emergency period.

Variable	Pre (%)	During (%)
***Anxiety/worry***	224 (48)	263 (57)
***Sadness/ discouragement***	191 (41)	225 (49)
***Fragility/vulnerability***	179 (39)	216 (47)
***Pessimism***	139 (30)	153 (33)
***Disorientation/confusion***	77 (17)	106 (23)
***Worsening of the quality of sleep***	135 (29)	165 (36)
***Lack of interest/pleasure***	364 (79)	344 (74)

**Fig 2:**
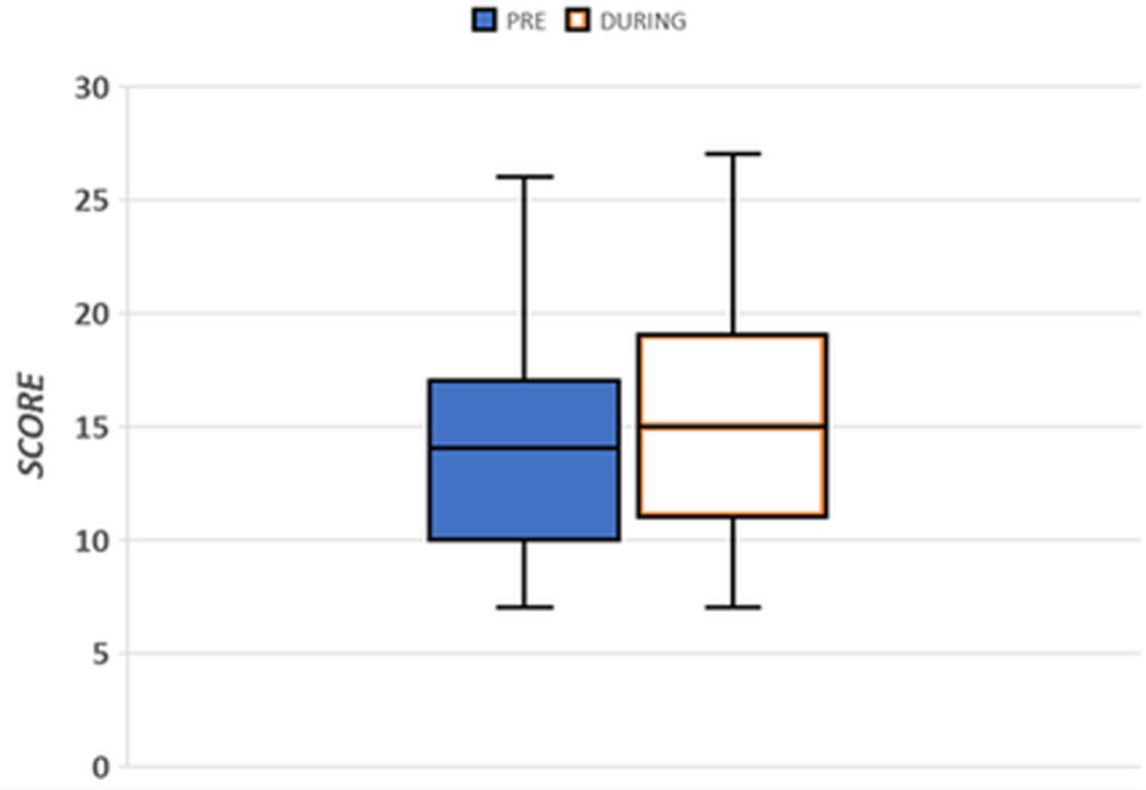
Quartile distribution of the casuistic.

The *EVI* increased during the pandemic to 210 (44%). An evaluation of each single item shows a certain degree of variability in the number of patients who gave a high score, from as low as 16% for the *feeling of disorientation/confusion* felt in the pre-pandemic phase (increased to 22% during the pandemic phase) to as high as 77% in the pre-pandemic phase (decreased to 73% during the pandemic phase) for the “*lack of interest/pleasure”*. **Fig 2** shows the quartile distribution of the casuistic.

**Fig 3:**
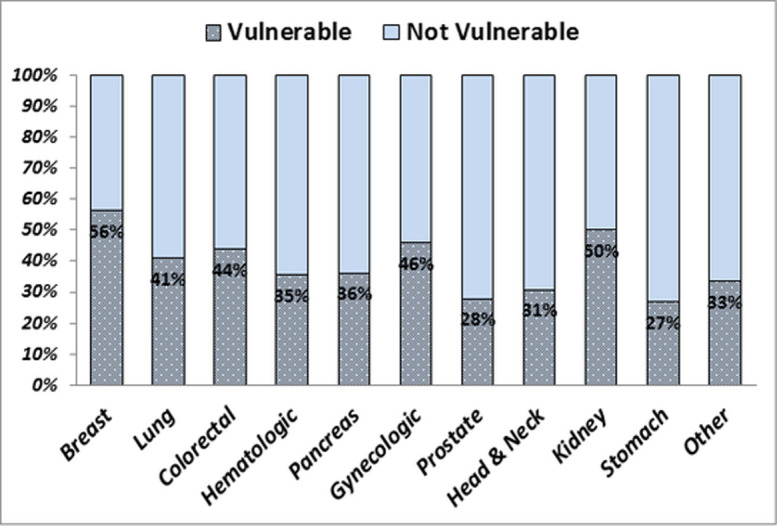
Emotional Vulnerability Index (EVI) and type of cancers.

Some degree of variability of the feeling of *EVI* was observed across the tumor types with the majority of breast cancer patients (56%) presenting a high score of vulnerability; this proportion falls among prostate (28%) and stomach (27%) cancers (**Fig 3**).

During the emergency period the feeling of low vulnerability remained stable for 216 (47%) while for 41 patients (9%) there was an increase. In 196 patients (42%) the feeling of vulnerability remained stably high and, surprisingly enough, in 10 patients (2%) their feeling of vulnerability was reduced.

Among the clinical characteristics (age, gender, ECOG performance status, clinical stage, therapy setting, therapy line, type of anticancer therapy) investigated in patients who were vulnerable in the pre-pandemic phase the only two who retained a statistically significant association with the likelihood of emotional vulnerability were female gender and chemotherapy (**Table 4**).

**Table 4:** Correlation between clinical variables and patients’ vulnerability in the pre-pandemic period.

PRE (SCORE ≥ 15)				
*Clinical variables*	*N*. *total*	*N*. *of vulnerable*	*Proportion (IC 95%)*	*P-value*
**AGE**				
**≤*65***	282	140	0.496 (0.439–0.554)	N.S.
***>65***	180	75	0.417 (0.347–0.490)	
**GENDER**				
***Female***	301	157	0.552 (0.465–0.577)	0.000923
***Male***	161	58	0.360 (0.290–0.437)	
**PERFORMANCE STATUS (ECOG)**				
***0–1***	433	199	0.460 (0.413–0.507)	N.S.
***≥2***	29	16	0.552 (0.376–0.715)	
**STAGE**				
***I-III***	242	116	0.479 (0.417–0.542)	N.S.
***IV***	220	99	0.450 (0.386–0.516)	
**THERAPY SETTING**				
***Neoadjuvant/Adjuvant***	242	116	0.479 (0.417–0.542)	N.S.
***Metastatic***	220	99	0.450 (0.386–0.516)	
**THERAPY LINE**				
***First line***	121	52	0.430 (0.345–0.519)	N.S.
***Subsequent lines***	99	47	0.475 (0.379–0.572)	
**ANTICANCER THERAPY**				
***Chemotherapy***	291	140	0.481 (0.424–0.538)	0.000152
***Biologicals***	156	44	0.282 (0.217–0.358)	

**Table 5** depicts the correlations of the feeling of vulnerability with the above mentioned variables during the Covid 19 pandemic in two hundred and forty six patients (53%) and shows that female gender and chemotherapy retained a significant association with the feeling of emotional vulnerability together with a young age (≤ 65 years).

**Table 5:** Correlation between clinical variables and patients’ vulnerability during the pandemic period.

DURING (SCORE ≥ 15)				
*Clinical variables*	*N*. *total*	*N*. *of vulnerable*	*Proportion (IC 95%)*	*P-value*
**AGE**				
**≤*65***	282	161	0.553 (0.513–0.627)	0.038126
***>65***	180	85	0.472 (0.401–0.545)	
**GENDER**				
***Female***	301	184	0.611 (0.555–0.665)	0,0000034
***Male***	161	62	0.385 (0.314–0.462)	
**PERFORMANCE STATUS (ECOG)**				
***0–1***	433	230	0.535 (0.484–0.578)	N.S.
***≥2***	29	16	0.552 (0.376–0.715)	
**STAGE**				
***I-III***	242	135	0.558 (0.495–0.619)	N.S
***IV***	220	111	0.505 (0.439–0.570)	
**THERAPY SETTING**				
***Neoadjuvant/Adjuvant***	242	135	0.558 (0.495–0.619)	N.S
***Metastatic***	220	111	0.505 (0.439–0.570)	
**THERAPY LINE**				
***First line***	121	59	0.488 (0.400–0.576)	N.S
***Subsequent lines***	99	52	0.525 (0.428–0.621)	
**ANTICANCER THERAPY**				
***Chemotherapy***	291	159	0.546 (0.489–0.603)	0.012938
***Biologicals***	156	66	0.423 (0.348–0.502)	

Female gender was the only variable to show a statistically significant association with the feeling of emotional vulnerability among the 41 patients whose feeling of vulnerability increased during the pandemic **(Table 6)**.

**Table 6:** Correlation between clinical variables and the increase of vulnerability during the pandemic period.

LOW to HIGH				
*Clinical variables*	*N*. *total*	*N*. *of vulnerable*	*Proportion (IC 95%)*	*P-value*
**AGE**				
**≤*65***	282	28	0.099 (0.069–0.140)	N.S.
***>65***	180	13	0.072 (0.042–0.121)	
**GENDER**				
***Female***	301	33	0.110 (0.079–0.151)	0.030856
***Male***	161	8	0.050 (0.024–0.097)	
**PERFORMANCE STATUS (ECOG)**				
***0–1***	433	40	0.068 (0.068–0.124)	N.S.
***≥2***	29	1	0.034 (0.000–0.189)	
**STAGE**				
***I-III***	242	27	0.112 (0.078–0.158)	N.S.
***IV***	220	14	0.064 (0.038–0.105)	
**THERAPY SETTING**				
***Neoadjuvant/Addjuvant***	242	27	0.112 (0.078–0.158)	N.S.
***Metastatic***	220	14	0.064 (0.038–0.105)	
**THERAPY LINE**				
***First line***	121	8	0.066 (0.032–0.128)	N.S.
***Subsequent lines***	99	6	0.061 (0.026–0.129)	
**ANTICANCER THERAPY**				
***Chemotherapy***	291	30	0.103 (0.073–0.144)	N.S.
***Biologicals***	156	9	0.058 (0.030–0.108)	

The ten patients who presented a reduction of the feeling of emotional vulnerability from high to low during the pandemic have an age ranging from 40 to 67 year (median 56) are mainly female (8/10) and are affected by breast cancer (4 patients) lung cancer (3 patients) ovarian, uterine and prostate cancer (one patient) treated with chemotherapy (6 patients), immunotherapy (3 patients) and target therapy (1 patient).

Finally, since it is conceivable that during phase 1 the *EVI*, could be higher than during the following phases, the 65 questionnaires (14%) collected during this phase (April 27^th^–May 03^rd^) were analyzed separately from the remaining 409 (86%) collected during Phase 2 (May 04^th^–June 07^th^) and the results show that during phase 1 patients were more likely to undergo the worsening of their vulnerability (10/65, 15.4%, as compared to 32/409, 0.8%; p = 0.006).

## Discussion

Cancer is a complex disease which encompasses several entities associated with peculiar biology, clinical history and evolution, stage at presentation and prognosis. Although it is usually difficult to convey all the complexity of the disease in normal situation the task can become overwhelming in a catastrophic situation such the Covid 19 pandemic when the idea that “cancer patients” in general are at a very high risk of severe complications and possibly of death, if infected, is widespread. Patients worry not only for the risk of getting infected but are also concerned of a possible neglect of their cure by a health system engulfed with Covid 19 patients. In this scenario the ability of patients to cope with the disease can be impaired and increased support by health personnel might be needed.

In this study involving a significant number of cancer patients under active treatment for different types of solid and hematologic cancers we wanted to study the impact of the Covid 19 pandemic on their “basal” psycho-social state through the administration of a simple questionnaire in which patients were asked to describe whether their feelings were changed in the “during the pandemic” period, as compared to the “pre-pandemic” period. We acknowledge that this method of survey brings the risk of recall bias. In fact the best way to conduct the survey would be through the administration of the same questionnaire before and during the pandemic but unfortunately the speed and the degree of destruction brought about by the pandemic did not allow this kind of evaluation.

Instead of evaluating the results of the questionnaires according to pre-defined scores, chosen arbitrarily, we decided to discriminate the level of “emotional vulnerability” shown by patients on the base of the observed median value of the score. As a result, we obtained two group of patients of almost the same consistency with a tiny majority of patients (247, 53%) falling in the low-score group and the remaining patients (215, 47%) falling in the high score group. Among the patients who are more vulnerable in a “basal” situation the clinical characteristic which showed a statistically significant association with the vulnerability are female gender and type of therapy (chemotherapy compared with other treatments). The questionnaires showed that this feeling of vulnerability increased during the pandemic in 41 patients and decreased in ten patients. As a results the patients vulnerable during the pandemic are 246 and again are more likely to be female and to be on treatment with chemotherapy. In this group the new variable age emerges as statistically significant, with patients ≤ 65 years being more emotionally vulnerable. The only clinical characteristic associated with the increased of the feeling of vulnerability in a statistically significant matter among the 41 patients who became more vulnerable during the pandemic is female gender.

The observation that female patients are more vulnerable is in line with results from several authors, suggesting an assumption of women’s higher vulnerability to the effects of stressful life events [**14–17**]. It is also conceivable that chemotherapy, more toxic compared to other forms of anti-cancer treatment present a heavier impact on the feeling of emotional vulnerability shown by patients.

In this study the psychological consequences of the pandemic were better handled by patients of 65 years of age or older, in line with previous reports showing that older cancer patients may present less psychological distress than younger patients **[18–20]**.

The negative influx of the pandemia on the vulnerability is corroborated by the observation that questionnaires administered during the phase 1, the closest to the phase of lockdown with its corollary of daily bad news and video of military trucks transporting dead bodies to the incineration facilities, were more likely to pick up an increased feeling of distress. Unfortunately, the study could be performed only in the final phases of the pandemic and this is for sure a point of weakness.

In conclusion, the pandemic did have an impact on the feeling of vulnerability shown by cancer patients especially among patients of female gender, patients 65 year old or younger and patients being treated with chemotherapy.

## Supporting information

S1 Questionnaire (DOCX)

S2 Questionnaire (DOCX)
