# Supplementary material for: Psychological impact of Covid-19 pandemic on oncological patients: A survey in Northern Italy
Source: PLoS One. 2021 Mar 16;16(3):e0248714. doi: 10.1371/journal.pone.0248714 (PMC7963060; doi:10.1371/journal.pone.0248714)
Supplement: S1 Questionnaire — (DOCX) [file pone.0248714.s001.docx]

| 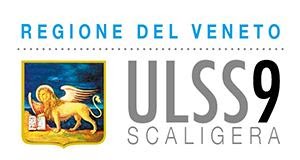  Sede Legale Via Valverde, 42 – 37122 Verona  cod.fisc. e P. IVA 02573090236  *DIPARTIMENTO DI ONCOLOGIA MEDICA*  *Direttore: Dott. Andrea Bonetti* | 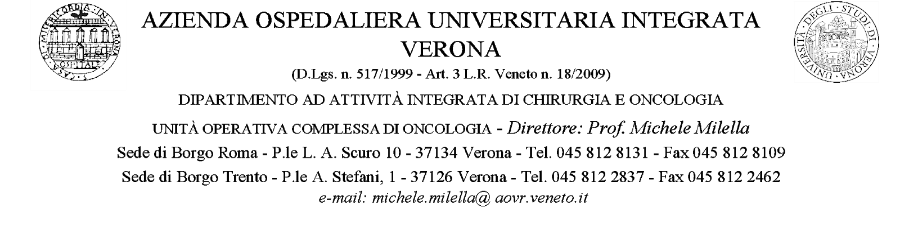 |
| --- | --- |

Dear Sir/Madame,

in this period of health emergency due to the Covid-19 pandemic, we would like to know what difficulties you are facing and how much this event is affecting you, both emotionally and regarding your illness.

Below is a list of questions that investigate the critical issues that people usually encounter in stressful moments in life.

We ask you to answer each question indicating how you are involved, using before and during the Covid-19 emergency as time references.

*Thank you for your collaboration*

| **ONCOLOGIST SECTION** |
| --- |
| ***Primary tumor:***  *□ Breast □ Lung □ Gastric □ Colorectal □ Ovary □ Head&Neck □ Hematology □ Other, specify: ­­­­­_________________* |
| ***Stage:***  *□ I □ II □ III □ IV* ***If stage IV, therapy line*** *□ 1° □ 2° □ 3° □ other* |
| ***Location:***  *□ AULSS9 Legnago □ AULSS9 San Bonifacio □ AULSS9 Villafranca □ AOUI Borgo Trento □ AOUI Borgo Roma* |

| **PATIENT SECTION**  **Questionnaire about emotional impact of Covid-19 in patients under anti-cancer treatment** | | | |
| --- | --- | --- | --- |
| Date of questionnaire completion ________________  Patient’s initials _____________  Year of birth ________________  Sex: *□* F *□* M  Marital status: *□* single *□* marital *□* in a domestic partnership *□* widower *□* other  School: *□* Elementary school *□* Secondary school graduate *□* High school graduate *□* Graduated  Job: *□* Unemployed *□* Employed *□* Other (student, job-seeker..) *□* Retired  Date first anticancer treatment ___________________  Diagnosis _______________________ | | | |
| **1. Were you positive to Covid-19?** | | | |
| *□* Yes *□* No *□* Not done  If yes, did you need assistance?: *□* Home care *□* Hospitalizazion  If yes, did you worry about your oncological disease?  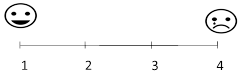  PER NULLA POCO ABBASTANZA MOLTO | | | |
| **2. Was a family member or friend positive to Covid-19?** | | | |
| *□* Yes *□* No  If yes, how was your mood?  _______________________________________________________________  ­­­­­­­­­­­­­­­­_______________________________________________________________  _______________________________________________________________ | | | |
| **3.** **How much do you feel anxious/worried for your cancer??** | | | |
| *Before Covid-19 pandemic*  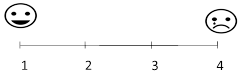  NOT AT ALL A LITTLE A QUITE A LOT | | *During Covid-19 pandemic*  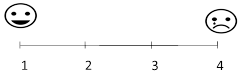  NOT AT ALL A LITTLE A QUITE A LOT | |
|  | | | |
| **4. How much do you feel sad/discouraged for your cancer?** | | | |
| *Before Covid-19 pandemic*  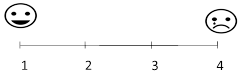  NOT AT ALL A LITTLE A QUITE A LOT | | *During Covid-19 pandemic*  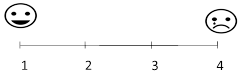  NOT AT ALL A LITTLE A QUITE A LOT | |
| **5. How much do you feel fragile/vulnerable for your cancer?** | | | |
| *Before Covid-19 pandemic*  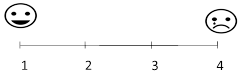  NOT AT ALL A LITTLE A QUITE A LOT | | *During Covid-19 pandemic*  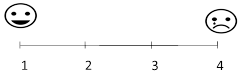  NOT AT ALL A LITTLE A QUITE A LOT | |
| **6. Are you pessimistic about the cure of your cancer?** | | | |
| *Before Covid-19 pandemic*  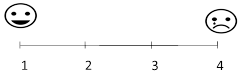  NOT AT ALL A LITTLE A QUITE A LOT | | *During Covid-19 pandemic*  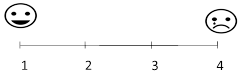  NOT AT ALL A LITTLE A QUITE A LOT | |
| **7. How much do you feel disoriented/confused about the management of your cancer?** | | | |
| *Before Covid-19 pandemic*  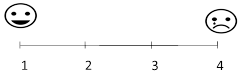  NOT AT ALL A LITTLE A QUITE A LOT | | *During Covid-19 pandemic*  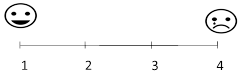  NOT AT ALL A LITTLE A QUITE A LOT | |
| **8. How much the concerns about your cancer influence the quality of your sleep?** | | | |
| *Before Covid-19 pandemic*  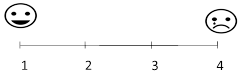  NOT AT ALL A LITTLE A QUITE A LOT | | *During Covid-19 pandemic*  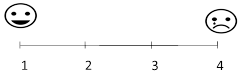  NOT AT ALL A LITTLE A QUITE A LOT | |
| **9. Do you feel pleasure for actions you have always enjoyed?** | | | |
| *Before Covid-19 pandemic*  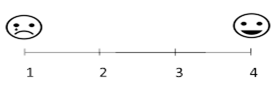  NOT AT ALL A LITTLE A QUITE A LOT | | *During Covid-19 pandemic*  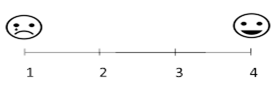  NOT AT ALL A LITTLE A QUITE A LOT | |
| **10. How much do you feel supported/helped in dealing with your cancer by your family members?** | | | |
| *Before Covid-19 pandemic*  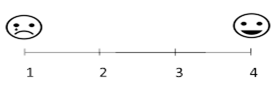  NOT AT ALL A LITTLE A QUITE A LOT | | *During Covid-19 pandemic*  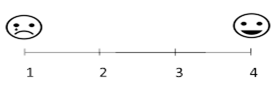  NOT AT ALL A LITTLE A QUITE A LOT | |
| **11. How much do you feel supported/helped in dealing with your cancer by the staff of the oncology center?** | | | |
| *Before Covid-19 pandemic*  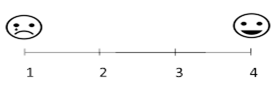  NOT AT ALL A LITTLE A QUITE A LOT | | *During Covid-19 pandemic*  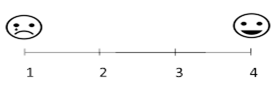  NOT AT ALL A LITTLE A QUITE A LOT | |
| **12. Overall, in this situation of generalized hardship due to Covid 19 emergency do you feel your discomfort increase?** | | | |
|  | | | |
| 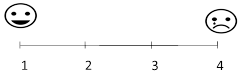  NOT AT ALL A LITTLE A QUITE A LOT  If yes, why?  _________________________________________________________________  _________________________________________________________________  _________________________________________________________________ | | | |
| **13. How much the Covid 19 emergency influences the management of your cancer?** | | | |
|  | | | |
| 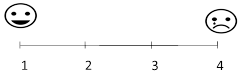  NO WAY A LITTLE A QUITE A LOT  If you answered a quite or a lot, which is the reason?   - Visits cancellation - Blood analysis cancellation - Services re-organization via telematic support (phone/skype) - Anti-cancer treatment change - Other (specify) ___________________________________ | | | |
