# Supplementary material for: Psychological impact of Covid-19 pandemic on oncological patients: A survey in Northern Italy
Source: PLoS One. 2021 Mar 16;16(3):e0248714. doi: 10.1371/journal.pone.0248714 (PMC7963060; doi:10.1371/journal.pone.0248714)
Supplement: S2 Questionnaire — (DOCX) [file pone.0248714.s002.docx]

| 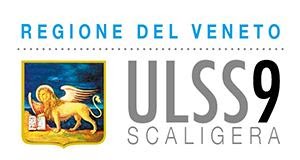  Sede Legale Via Valverde, 42 – 37122 Verona  cod.fisc. e P. IVA 02573090236  *DIPARTIMENTO DI ONCOLOGIA MEDICA*  *Direttore: Dott. Andrea Bonetti* | 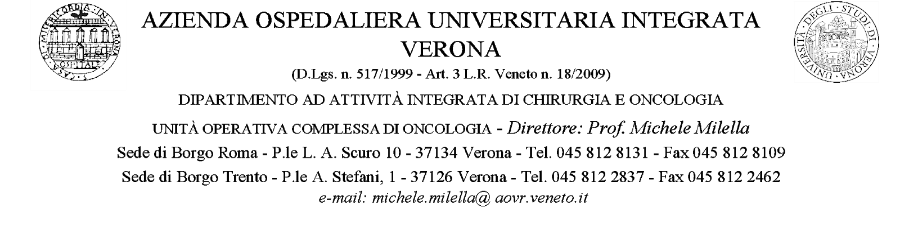 |
| --- | --- |

Buongiorno Signora/Signore,

in questo periodo di emergenza sanitaria dovuta alla pandemia di Covid-19, vorremmo conoscere quali difficoltà Lei sta affrontando e quanto questo evento La stia influenzando, sia emotivamente che riguardo la sua malattia.

Di seguito è presente una lista di domande che indagano le criticità che solitamente le persone incontrano nei momenti stressanti della vita.

Le chiediamo di rispondere a ciascuna domanda indicando quanto ne sia coinvolta/o, utilizzando come riferimenti temporali il prima e durante l’emergenza Covid-19.

*Grazie per la collaborazione*

| **SEZIONE A CURA DELL’ONCOLOGO** |
| --- |
| ***Sede di malattia primitiva:***  *□ Mammella □ Polmone □ Stomaco □ Colon-retto □ Ovaio □ Capo-collo □ Ematologico □ Altro, specificare: ­­­­­_________________* |
| ***Stadio di malattia:***  *□ I □ II □ III □ IV* ***In caso di stadio IV, indicare*** ***linea di terapia:*** *□ 1° □ 2° □ 3° □ successive* |
| ***Sede di cura:***  *□ AULSS9 Legnago □ AULSS9 San Bonifacio □ AULSS9 Villafranca □ AOUI Borgo Trento □ AOUI Borgo Roma* |

| **SEZIONE A CURA DEL PAZIENTE**  **Questionario di valutazione dell’impatto emotivo del Covid-19 su pazienti in trattamento oncologico** | | | |
| --- | --- | --- | --- |
| Data di compilazione del questionario ________________  Iniziali del paziente _____________  Anno di nascita ________________  Genere: *□* F *□* M  Stato civile: *□* nubile/celibe *□* sposata/o *□* convivente *□* vedova/o *□* altro  Titolo di studio: *□* nessuno *□* licenza scuola media *□* diploma superiore *□* laurea  Occupazione: *□* non occupata/o *□* lavoratrice/ore *□* in cerca di occupazione *□* pensionata/o *□* altro  In cura da (indicare il tempo della presa in carico oncologica) ___________________  Diagnosi oncologica _______________________ | | | |
| **1. E’ risultata/o positiva/o al tampone per il Covid-19?** | | | |
| *□* Si *□* No *□* Non l’ho fatto  Se ha risposto Si, indichi qual è stato il suo bisogno assistenziale: *□* seguito a casa *□* ricovero ospedaliero  Se ha risposto **Sì**, indichi il suo stato di preoccupazione in relazione alla sua malattia oncologica  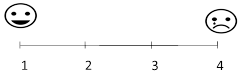  PER NULLA POCO ABBASTANZA MOLTO | | | |
| **2. Qualcuno della sua rete familiare/amicale è risultato positivo al tampone per il Covid-19?** | | | |
| *□* Si *□* No  Se Sì, può descrivere qual è/è stato il suo stato d’animo a riguardo?  _______________________________________________________________  ­­­­­­­­­­­­­­­­_______________________________________________________________  _______________________________________________________________ | | | |
| **3.** **Quanto si sente in ansia/preoccupato per l’andamento della malattia oncologica?** | | | |
| *Prima dell’emergenza Covid-19*  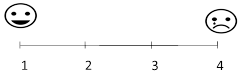  PER NULLA POCO ABBASTANZA MOLTO | | *Durante l’emergenza Covid-19*  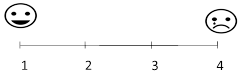  PER NULLA POCO ABBASTANZA MOLTO | |
|  | | | |
| **4. Quanto si sente triste/sconfortato in relazione alla malattia oncologica?** | | | |
| *Prima dell’emergenza Covid-19*  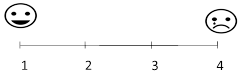  PER NULLA POCO ABBASTANZA MOLTO | | *Durante l’emergenza Covid-19*  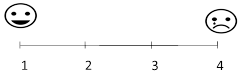  PER NULLA POCO ABBASTANZA MOLTO | |
| **5. Quanto si sente fragile/vulnerabile in relazione alla malattia oncologica?** | | | |
| *Prima dell’emergenza Covid-19*  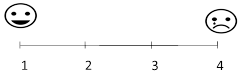  PER NULLA POCO ABBASTANZA MOLTO | | *Durante l’emergenza Covid-19*  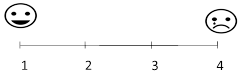  PER NULLA POCO ABBASTANZA MOLTO | |
| **6. Quanto si sente pessimista riguardo la guarigione/cura della malattia oncologica?** | | | |
| *Prima dell’emergenza Covid-19*  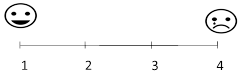  PER NULLA POCO ABBASTANZA MOLTO | | *Durante l’emergenza Covid-19*  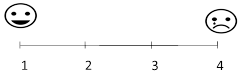  PER NULLA POCO ABBASTANZA MOLTO | |
| **7. Quanto si sente disorientato/confuso per la gestione della malattia oncologica?** | | | |
| *Prima dell’emergenza Covid-19*  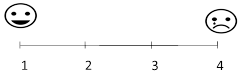  PER NULLA POCO ABBASTANZA MOLTO | | *Durante l’emergenza Covid-19*  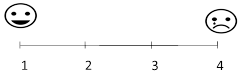  PER NULLA POCO ABBASTANZA MOLTO | |
| **8. Quanto le preoccupazioni legate alla malattia oncologica influenzano la qualità del suo sonno (addormentamento/risvegli)?** | | | |
| *Prima dell’emergenza Covid-19*  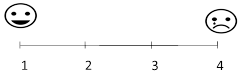  PER NULLA POCO ABBASTANZA MOLTO | | *Durante l’emergenza Covid-19*  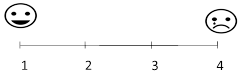  PER NULLA POCO ABBASTANZA MOLTO | |
| **9. Riesce a provare piacere per le cose che ha sempre fatto volentieri?** | | | |
| *Prima dell’emergenza Covid-19*  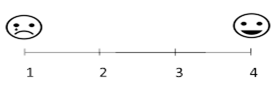  PER NULLA POCO ABBASTANZA MOLTO | | *Durante l’emergenza Covid-19*  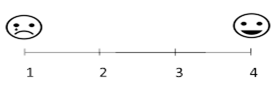  PER NULLA POCO ABBASTANZA MOLTO | |
| **10. Quanto si sente supportato/aiutato nella gestione della malattia oncologica dai suoi familiari?** | | | |
| *Prima dell’emergenza Covid-19*  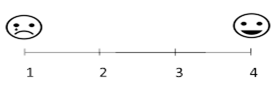  PER NULLA POCO ABBASTANZA MOLTO | | *Durante l’emergenza Covid-19*  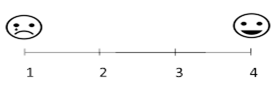  PER NULLA POCO ABBASTANZA MOLTO | |
| **11. Quanto si sente sostenuto/aiutato nella cura della malattia oncologica dal Servizio di oncologia a cui afferisce?** | | | |
| *Prima dell’emergenza Covid-19*  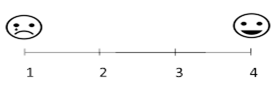  PER NULLA POCO ABBASTANZA MOLTO | | *Durante l’emergenza Covid-19*  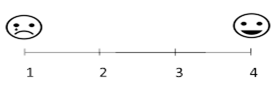  PER NULLA POCO ABBASTANZA MOLTO | |
| **12. Complessivamente, in questa situazione di disagio generale legata all’emergenza Covid-19,**  **il suo stato di malessere, lo percepisce aumentato?** | | | |
|  | | | |
| 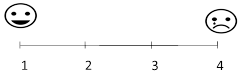  PER NULLA POCO ABBASTANZA MOLTO  Se ha risposto abbastanza o molto alla domanda 10 può indicarne i motivi?  _________________________________________________________________  _________________________________________________________________  _________________________________________________________________ | | | |
| **13. In relazione all’emergenza Covid-19 quanto è cambiata la gestione della sua malattia?** | | | |
|  | | | |
| 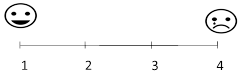  PER NULLA POCO ABBASTANZA MOLTO  Se ha risposto abbastanza o molto alla domanda 13 può indicarne i motivi?   - annullamento di visite mediche - annullamento di esami - ri-organizzazione dei servizi con modalità telematiche (telefono/skype) - cambiamento di trattamento farmacologico - Altro (specificare) ___________________________________ | | | |
